# Use of the Spare-Part Strategy to Reconstruct the External Auditory Canal After Subtotal Auriculectomy for Basal Cell Carcinoma

**DOI:** 10.1007/s12070-021-02654-1

**Published:** 2021-05-22

**Authors:** Marco Pignatti, Gioia Sorbi, Valentina Pinto, Giovanni Sorrenti, Riccardo Cipriani

**Affiliations:** 1 Plastic Surgery, IRCCS – Azienda Ospedaliero-Universitaria di Bologna, Via Albertoni 15, 40138 Bologna, Italy; 2 DIMES-Università di Bologna, Alma Mater Studiorum, Bologna, Italy; 3 Plastic Surgery, Policlinico di Modena, University of Modena and Reggio Emilia, Modena, Italy; 4 Otolaryngology Head and Neck Surgery, IRCCS – Azienda Ospedaliero-Universitaria di Bologna, Bologna, Italy

**Keywords:** Subtotal auriculectomy, Basal cell carcinoma, Local flap, Spare-part surgery

## Abstract

After removal of an infiltrative BCC of the auditory meatus, a soft tissue defect of the temporal-mastoid area with bone exposure, needed reconstruction. Several options have been taken into account and a simple yet effective solution has been found following the spare-parts principle. The ear lobe, preserved during cancer removal, was split and used as a thin skin flap. Adequate coverage of the bone exposure and resurfacing of the external auditory canal was obtained with minimal donor site morbidity and a short surgery in a fragile patient with several comorbidities. The spare-parts strategy can provide successful solution to difficult reconstructive cases regardless of the anatomical area.

## Case Report

An 82-year-old Caucasian man presented with a biopsy-proven retro-auricular basal cell carcinoma (BCC) affecting the right auricle and the external auditory meatus (EAM).

Craniofacial contrast-enhanced Computed Tomography showed that the lesion was reaching the tympanic membrane and eroding the bone of the antero-inferior wall of the EAM. The tumor was in contact with the parotid gland and with the mandibular condyle, but did not invade the bone.

**Fig. 1:**
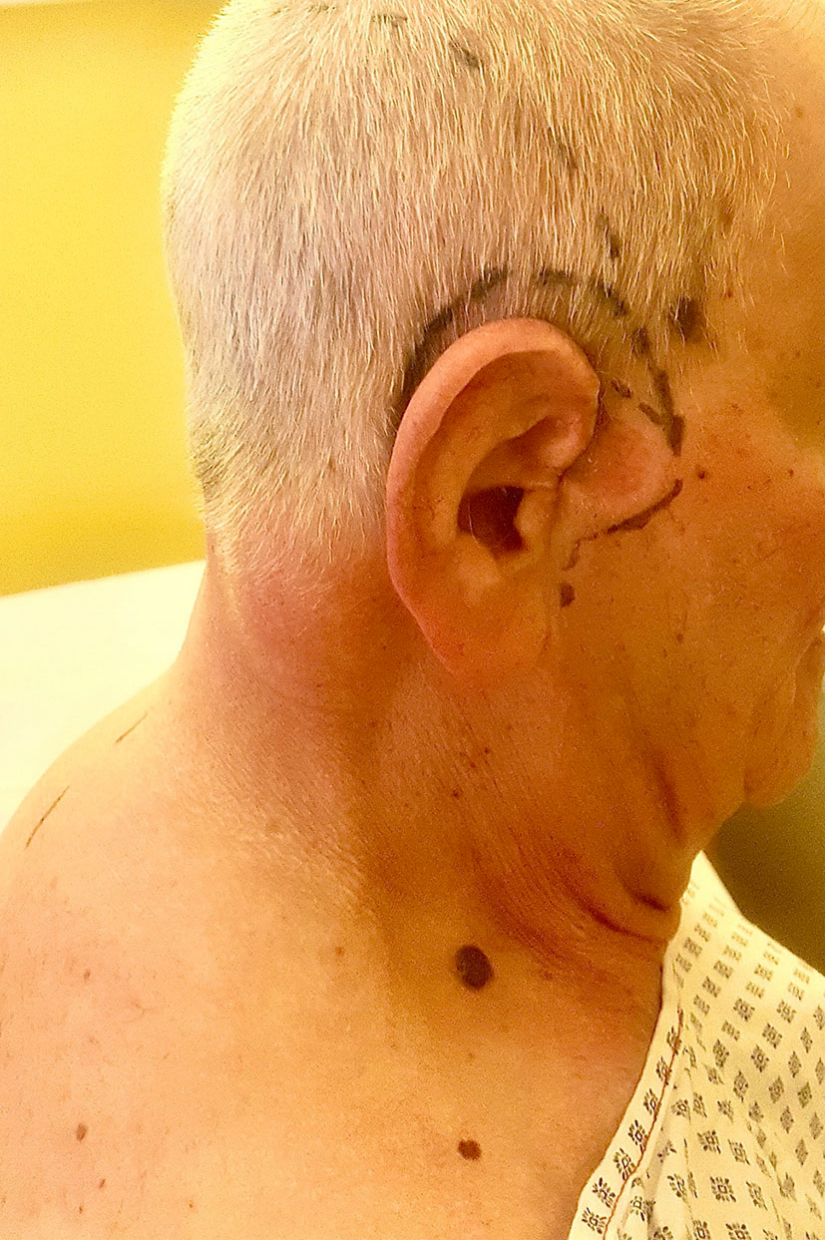
BCC of the external ear. Preoperative markings maintaining an excision margin of 1 cm

Surgery was planned for tumor removal with clear margins of 1 cm from the macroscopically visible BCC borders followed by immediate soft tissue reconstruction (Fig. 1).

Tumor clearance required en-bloc resection of the auricle (except the lobe) and a small superficial portion of the posterior pole of the parotid, down to the temporalis muscle and the bone of the EAM.

Finally, skin removal and bone milling of the walls of the EAM were performed, preserving the tympanic membrane.

**Fig. 2:**
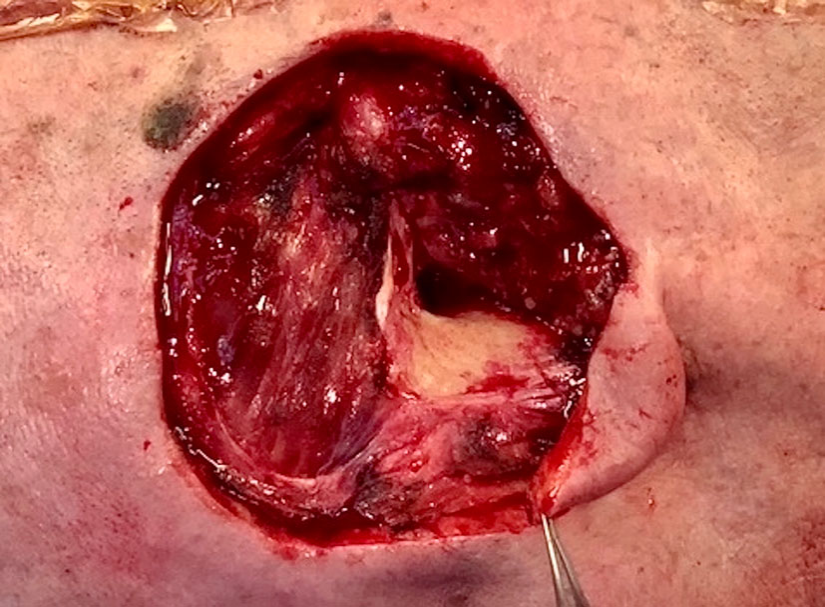
Residual defect after tumor removal: mastoid bone, ear canal, superficial parotid gland, and posterior part of temporalis muscle are exposed. (Patient supine. The left border of the picture is cranial)

Cancer removal resulted in an 8 by 7 cm defect exposing the posterior portion of the temporalis muscle and an area of 2.5 by 1.5 cm of the temporal bone, not covered by periosteum, posteriorly to the EAM (Fig. 2).

Several surgical options were considered as discussed below. The factors to be evaluated in deciding the reconstructive choice were: the patient's comorbidities (chronic obstructive pulmonary disease, previous silent heart attacks, bilateral carotid stenosis) requiring a short surgery, the need to provide bone coverage while preserving the auditory canal patency,.

To obtain these results, following the "spare parts" strategy [1], we planned a unique local flap, obtained from the ear lobe preserved during tumor removal.

The essence of this concept, frequently used in limb reconstruction, is to always consider the reconstructive potential of the discarded and apparently useless tissues in the proximity of a defect.

**Fig. 3:**
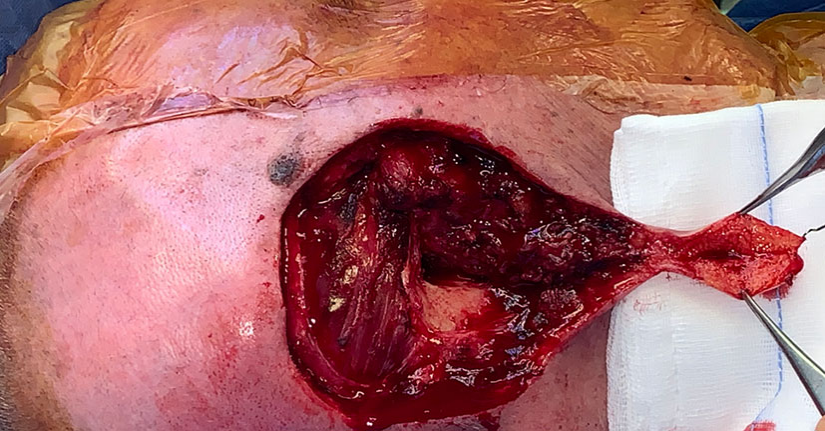
The ear lobe, speared in the cancer removal, becomes a flap: it is split to increase the skin surface and reduce its thickness while maintaining adequate perfusion

To obtain the maximal advantage from the available tissue, we split the ear lobe, while preserving the subcutaneous nourishing vessels (Fig. 3). The increased skin surface and the reduced thickness so obtained allowed us to cover the exposed mastoid bone and to resurface part of the ear canal.

A split-thickness skin graft from the thigh was used to cover the temporal muscle that was still exposed.

Finally, the splinting device NasoPore (Stryker, USA) was inserted in the ear canal to maintain the flap adherent to the bone during the initial healing period therefore preserving the patency of the ear canal.

Pathology revealed an infiltrative basal cell carcinoma, (an aggressive histologic subtype) with perineural infiltration. The excision margins were clear from cancer. After surgery, the patient underwent radiation adjuvant therapy [2] because of the large size of the tumor, its histologic features, and the extensive neural infiltration. Also, BCCs that impinge upon or invade the external auditory canal are associated with a higher risk of local recurrence [3].

**Fig. 4:**
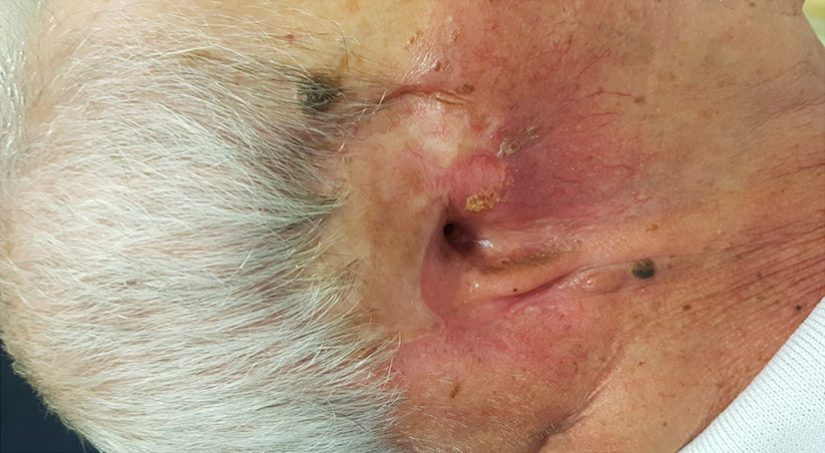
Six months follow-up: a silicone splint has helped to maintain EAM patency during healing

Follow-up at 6 months showed no clinical or radiological signs of tumor recurrence, nor skin damage due to radiotherapy. A satisfactory aesthetic and functional result, as well as the preservation of hearing, were obtained with minimal donor site morbidity (in relation to the skin graft harvest) (Fig. 4).

## Discussion

BCC of the external ear is common but usually of small size and limited to the skin. When infiltrative, it may pose difficult treatment challenges given the complexity of the local anatomy and the proximity to other noble structures, such as the parotid gland, the external auditory canal, the tympanic membrane, the middle ear, and the facial nerve.

We evaluated several reconstructive options that offered, despite the bone exposure, a simple solution, while maintaining the auditory canal open to preserve hearing.

Regional pedicled myo-cutaneous flaps, such as the pectoralis major flap and trapezius flap are usually bulky and might have had length limitations in reaching the tumor's site [4].

Furthermore, donor site morbidity is considerable and the coverage obtained would have obliterated the auditory canal.

Another option could have been the supraclavicular artery island flap, which causes less donor site morbidity but similar difficulties in preserving an open EAM [5].

The same problems are associated with free tissue transfer, which also requires a longer and more complex surgery.

A pedicled temporalis muscle flap with skin graft was planned as our first choice, due to its proximity, good perfusion, ease of harvest, and limited morbidity.

The muscle would have nicely covered the bone exposure but with the disadvantage of occluding the meatus.

On the contrary, local skin flaps, if thin enough, would have allowed also a resurfacing of the external meatus and canal.

No suitable local skin flaps were available in the area except for the remnants of the auriculectomy: the ear lobe.

We, therefore, applied the "spare-part" strategy, initially proposed for the reconstructive surgery of limbs, to the head and neck area.

According to this method, residual tissues after trauma or surgical resection may be used to reconstruct the missing tissues [6].

Its advantages are the simplicity and the limited donor site morbidity, while the disadvantages may be the amount and quality of the tissue available. Perfusion and function of the spare parts should always be verified before using them for reconstruction, especially after trauma or surgical incisions.

## Conclusions

We reported here the use of a portion of auricle as a flap for reconstruction. The tissue, being within safe margins, was preserved during cancer removal.

The use of scavenged tissue is referred to as "spare-part surgery", and it is an important reconstructive strategy in complicated cases.
